# How Eye Movements Stabilize Posture in Patients With Bilateral Vestibular Hypofunction

**DOI:** 10.3389/fneur.2018.00744

**Published:** 2018-09-18

**Authors:** Michel Lacour, Nadine Yavo Dosso, Sylvie Heuschen, Alain Thiry, Christian Van Nechel, Michel Toupet

**Affiliations:** ^1^Aix-Marseille University, Research Federation 3C, UMR CNRS 7260, Marseille, France; ^2^Service ORL CCF, CHU de Treichville, Abidjan, Côte d'Ivoire; ^3^Centre d'explorations Fonctionnelles Otoneurologiques, Paris, France; ^4^Rééducateur Vestibulaire, Nice, France; ^5^Unité Vertiges et Troubles de l'Equilibre, CHU Brugmann, Brussels, Belgium; ^6^Otolaryngology Department, Dijon University Hospital, Dijon, France

**Keywords:** bilateral vestibular hypofunction patients, static posturography, darkness, visual fixation, slow eye movements, saccades

## Abstract

Chronic patients with bilateral vestibular hypofunction (BVH) complain of oscillopsia and great instability particularly when vision is excluded and on irregular surfaces. The real nature of the visual input substituting to the missing vestibular afferents and improving posture control remains however under debate. Is retinal slip involved? Do eye movements play a substantial role? The present study tends to answer this question in BVH patients by investigating their posture stability during quiet standing in four different visual conditions: total darkness, fixation of a stable space-fixed target, and pursuit of a visual target under goggles delivering visual input rate at flicker frequency inducing either slow eye movements (4.5 Hz) or saccades (1.2 Hz). Twenty one chronic BVH patients attested by both the caloric and head impulse test were examined by means of static posturography, and compared to a control group made of 21 sex-and age-matched healthy participants. The posturography data were analyzed using non-linear computation of the center of foot pressure (CoP) by means of the wavelet transform (Power Spectral Density in the visual frequency part, Postural Instability Index) and the fractional Brownian-motion analysis (stabilogram-diffusion analysis, Hausdorff fractal dimension). Results showed that posture stability was significantly deteriorated in darkness in the BVH patients compared to the healthy controls. Strong improvement of BVH patients' posture stability was observed during fixation of a visual target, pursuit with slow eye movements, and saccades, whereas the postural performance of the control group was less affected by the different visual conditions. It is concluded that BVH patients improve their posture stability by (1) using extraocular signals from eye movements (efference copy, muscle re-afferences) much more than the healthy participants, and (2) shifting more systematically than the controls to a more automatic mode of posture control when they are in dual-task conditions associating the postural task and a concomitant visuo- motor task.

## Introduction

Balance control in healthy subjects is the result of the integration of multisensory signals originating from three spatial references: the allocentric (vision), the egocentric (somatosensory), and the geocentric (vestibular) spatial frames. It is known since a long time that posture stability is increased with eyes open compared to eyes closed, with vision of stable space-fixed target or with full-field vision compared to darkness (1). The real nature of the visual cues for posture stabilization remains however an open debate. During quiet standing, in non-challenging conditions, fixation or pursuit of a visual target modulates balance control. Among the afferent signals inducing the visually evoked postural effects is the retinal slip, i.e., the motion of the visual images on the retina (2). On the other hand, many studies showed that eye movements *per se* modify the postural performance. Posturography recordings showed postural stability improvement during saccadic tasks compared to fixation tasks in both children (3) and adults (4–7). Execution of eye movements was however reported to decrease postural stability when healthy participants pursuit a moving target in darkness and use smooth pursuit instead of saccades (8). Explanations from the literature indicate that posture stability changes during the visual task are due either to two different modes of detection of body sway, ocular vs. extraocular (2, 8), or to sharing of the attentional resources as an effect of dual tasking condition [postural task and eye movement task; (7)].

Bilateral vestibular hypofunction (BVH) is a disorder with different clinical pictures (combined or isolated deficits of the otolith and semicircular canal functions), and it remains a diagnostic challenge since there is still no consensus about diagnostic criteria (9). Reduced or absent function of the vestibular organs and/or vestibular nerves results in impairment or loss of the major vestibular functions: posture and balance control, gaze stabilization and spatial orientation (10). Indeed, spatial disorientation, oscillopsia, and balance problems are the main deficits reported by patients with BVH, and particularly in darkness and on irregular surfaces. Vestibular rehabilitation therapy helps those patients to regain an acceptable quality of life and, when examined in the light on regular support surfaces, they show not so dramatic postural performances. Perception of the visual vertical and perception of body tilt differed only marginally in bilateral a-reflexic patients compared to age-matched controls (11). Postural equilibrium on a stable platform was improved significantly with vision (12) and light touch from a fingertip (13, 14). Augmenting sensory information by providing auditory, visual and vibrotactile bio-feedback of body sway improved also the postural performance of BVH patients (15). All the data indicate that extra-vestibular signals substitute for vestibular input in BVH patients' spatial orientation, perception and posture control (16–18). Among the main sensory inputs substituting for the missing vestibular afferents in chronic bilateral vestibular failure are however the visual cues (19, 20).

In the present study, we tried to determine how the visual input can stabilize the postural performance of chronic BVH patients. A dynamic approach with a stable force platform was used to investigate the patients' posture control system. Recordings of the Center of foot Pressure (CoP) were made during quiet standing in four conditions: eyes open in total darkness, with vision of a stable space-fixed target, and during pursuit of a moving visual target in stroboscopic light with either high (4.5 Hz) or low (1.20 Hz) flicker frequency, which called into play the smooth pursuit system and the saccadic system, respectively. Data processing of the CoP displacements was performed with the wavelet transform and the fractional Brownian-motion analysis, which constitute powerful functional describers of posture control compared to conventional methods based only on posture parameters not enough sensitive [CoP length and area, for example: see (21)].

Considering the powerful role of vision in case of BVH, we hypothesized that chronic BVH patients would use all available visual cues, provided by stable space-fixed targets as well as tracking eye movements, as substitution strategies to improve their posture stability.

## Methods

### Subjects

Twenty one patients (11 female, 10 male, mean age: 62.9 years; range: 38–80 years) with BVH were included in the experiment. The history of the BVH patients showed that the disease was diagnosed several years ago (8 years on average), and that all patients had been followed by physiotherapists for intense vestibular rehabilitation therapy. When seen for the first time by the ENTs, they still complained of oscillopsia during fast head motion, with particular difficulty to read while walking, and of balance problems in the dark or on irregular surfaces. BVH was mostly idiopathic (71% of the patients). Four patients had BVH due to antibiotic ototoxicity, while bi-lateralization of Menière's disease was reported in two others. Finally, one patient reported an acute unilateral peripheral vestibular loss followed later on by another attack on the other side. Only one patient exhibited a remaining spontaneous vestibular nystagmus in the light (4.2°/s). Complete or sub-total loss of vestibular function was assessed mainly on the basis of two clinical tests: the caloric and the video head impulse test. The horizontal semicircular canal function was tested in the very low frequency part (0.003 Hz) with the caloric test (30 s irrigation of 150–200 cm^3^ at 30 and 44°C). All the BVH patients showed no responses to caloric irrigation or with slow phase eye velocity in the range 0–5°/s. The total sum for the four caloric tests was <20°/s, a criteria well defining BVH according to Vibert et al. (22). In the high velocity range (video Head Impulse Test: 10° amplitude; 200°/s), the horizontal and vertical canal functions of all the BVH patients showed reduced gains of the vestibulo-ocular reflexes (<0.5) and presence of both overt and covert saccades. The otolith function was tested by recording of the vestibular evoked myogenic potentials elicited by short tone bursts (95 dB; 500 Hz) and surface EMG electrodes at the sternocleidomastoid muscles (cVEMPs). Six BVH patients showed normal cVEMPs while three others exhibited higher threshold responses on both sides. The total lack of saccular responses was observed in two patients only and normal cVEMPs were observed on one side only in the remaining BVH patients. The oVEMPs have not been evaluated in our BVH population. The whole of the vestibular examinations indicate therefore that our population of BVH patients had absent or significantly reduced canal function in both the low and high frequency ranges, but most of them still exhibited remaining otolithic function. The data collected in the BVH patients were compared to those recorded in a control group made of 21 sex- and age-matched healthy participants (11 female, 10 male, mean age: 58.3 years; range: 32–82 years). The vestibular clinical tests have not been performed in the controls who, however, reported to be free of vestibular disorders.

All the patients and healthy controls provided informed consent before their participation. The experimental protocol was approved by the local Ethics Committee (CCPPRB Paris) and followed the recommendations of the Helsinki declaration.

### Experimental setup

Static posturography was done with a force-measuring platform (Multitest Equilibre, Framiral, Grasse, France) that records the CoP displacements (sampling frequency: 50 Hz; analog-digital converter: 16 bits) during sequences of 30 s. Patients were required to stand quietly, arms along the body, feet in natural position, and to stand as stable as possible without voluntary movements of head and body during the whole recording sessions. They were tested first with eyes open in total darkness (Dark), being instructed to look straight ahead an imaginary target located at eye level. During this recording session, and in order to reduce anxiety and stress, patients were aware that somebody located behind would take care and avoid any possible fall. In a second session, the patients had to perform the same postural task while fixating in total darkness a visual red target (Fixation) located at eye level, 1.2 m in front of them (lateral field of view: 95°). For the third and fourth sessions, the patients were instructed to follow with the eyes the visual target which was moving sinusoidally in the horizontal plane at eye level (25 degrees amplitude; 0.13 Hz frequency). During these recording sessions the patients were equipped with stroboscopic goggles, mounted on the videonystagmography goggles, that provided light flashes at high frequency (strob 1 condition: flicker frequency of 4.5 Hz; flash duration: 100 ms; 3rd session) or low frequency (strob 2 condition: flicker frequency of 1.20 Hz; flash duration: 200 ms; 4th session). Continuous perception being observed with flicker frequency higher than 4 Hz (1), slow eye movements (smooth pursuit) were done by the patients to pursuit the visual target at the high flicker frequency (Strob 1 condition), whereas saccades were observed at the low flicker frequency (Strob 2 condition) for which only partial visual stabilization was possible. Eye movements were recorded by video-oculography (Framiral, Grasse, France; lateral field of view of goggles: 50°). Calibration of the eye movements was performed at the beginning of the recordings, when the patients were standing on the platform. It consisted of red sled targets (diameter: 0.5°) presented randomly at opposite locations on a sleds bar. The four visual conditions were performed in a total dark room to avoid fixation of other stimuli, and the order of the visual tasks was not varied randomly across patients.

**Figure 1:**
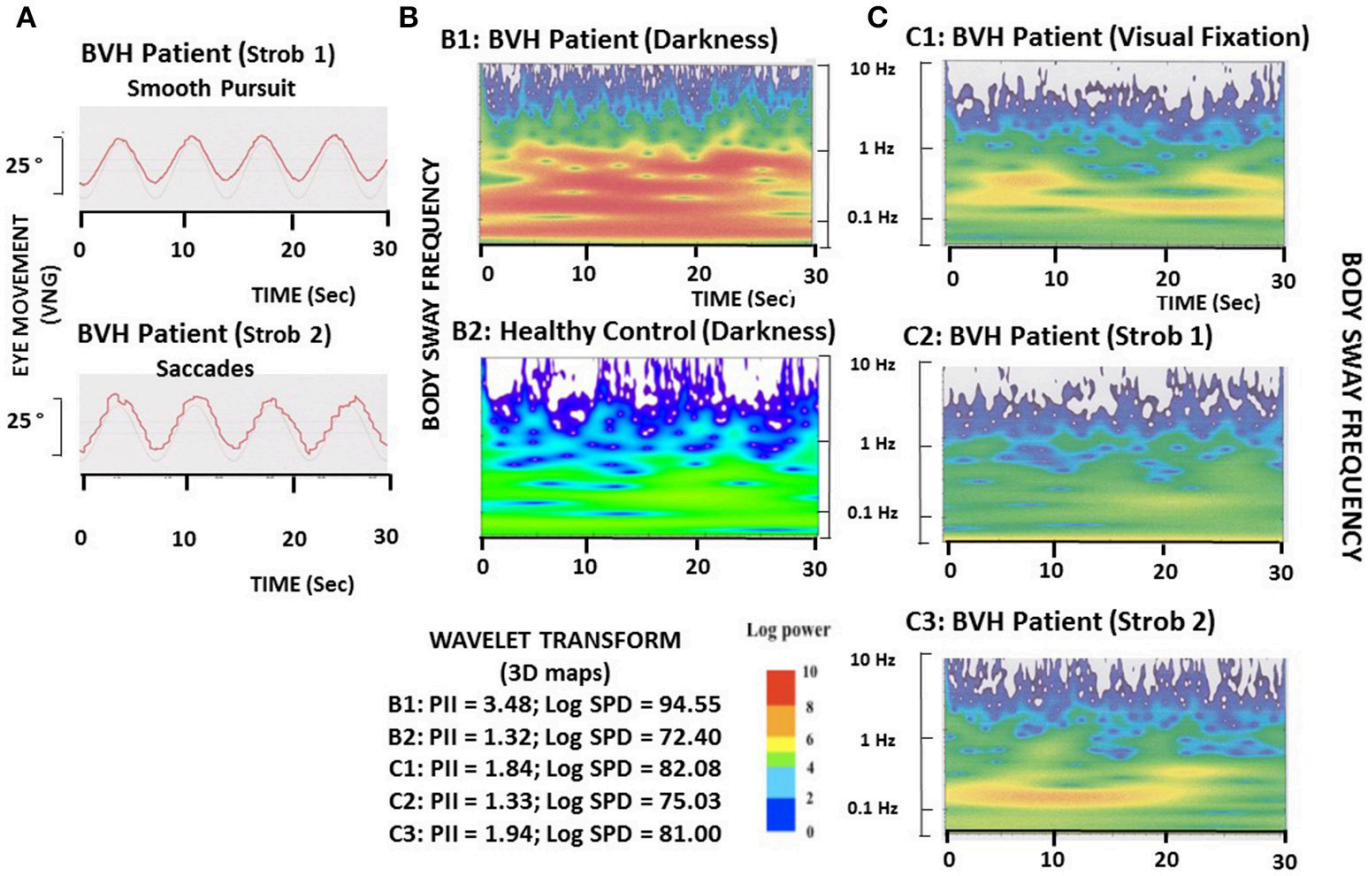
Illustration of eye movements and postural performance in one representative BVH patient during the different visual conditions. **(A)**. The BVH patient must pursuit a visual target moving sinusoidally in the horizontal plane (25° amplitude; 0.13 Hz frequency) in stroboscopic light at either the 4.5 Hz flicker frequency, which elicits slow eye movements (smooth pursuit: strob 1), or the 1.2 Hz flicker frequency, which induces saccadic eye movements (strob 2). **(B)**. Comparison of the wavelet transform applied on the antero-posterior CoP displacements between the BVH patient **(B1)** and one representative healthy subject **(B2)** tested in total darkness. The wavelet analysis provides a 3D chart of body sway with time on the abscissae (in seconds), frequency content of the stabilogram on the ordinates (log scale in Hz), and spectral power density shown as a color code for the third dimension (expressed in decimal log). The Postural Instability Index (PII) and the spectral power density (PSD, expressed in decimal log) in the frequency range of the visual system (0.05–0.5 Hz), derived from the wavelet plots, show both higher values in the BVH patient (3.48 and 94.55, respectively) compared to the healthy control (1.32 and 72.40, respectively). **(C)**. 3D chart of the antero-posterior sway of the BVH patient tested during visual fixation of the target **(C1)**, tracking of the target with slow eye movements (**C2**: strob 1), and saccadic pursuit (**C3**: strob 2). Compared to the recording made in total darkness (**B1**), fixation, smooth pursuit, and saccades improve the BVH patient's postural stability, as shown by the strong reduction of the PII and SPD (1.84 and 82.08, 1.33 and 75.03, and 1.94 and 81.00 for the three visual conditions, respectively).

The Figure 1A shows the typical slow eye movements (strob 1 condition: smooth pursuit) and the saccadic eye movements (strob 2 condition: saccades) recorded in one representative patient at the high and low flicker frequencies, respectively.

### Data processing

The CoP displacements were computed in the antero-posterior (AP) and medio-lateral (ML) directions and used to measure the postural performance of the patients. A non-linear analysis of CoP displacements was performed in order to accurately evaluate the posture control system [see (21), for review]. It consisted of applying both the wavelet transform and the fractional Brownian-motion analysis (PosturoPro software, Framiral) to the AP and ML stabilograms.

The wavelet analysis consisted of describing body sway frequencies as a function of time, a method we have described in details in our previous papers (21, 23, 24). This method has not the limitations of the Fast-Fourier Transform and provides a time-frequency chart of body sway in three dimensional space, giving access to the changes in the body sway frequency components with time. The spectral power density was expressed as a decimal logarithm scale reported on the 3D map by a color code (cf Figures 1B,C). The spectral power density (SPD) contains in the whole signal as well as in the frequency part corresponding to the main contribution of vision to postural regulation (0.05–0.5 Hz) were calculated separately for the CoP displacements in the AP and ML directions. The SPD parameter is also a good estimate of the energy cost required to maintain a stable postural performance. The Postural Instability Index (PII) derived from the wavelet plots was evaluated also [see (25)]. It was calculated from both the spectral power density contained in the whole stabilogram and the time during which the spectral power of the different body sway frequencies tend to be close to zero (cancellation time) by the close-loop control mechanisms [see (21)]. The PII was computed as a global score, independently of the AP or ML directions of the CoP displacements.

**Figure 2:**
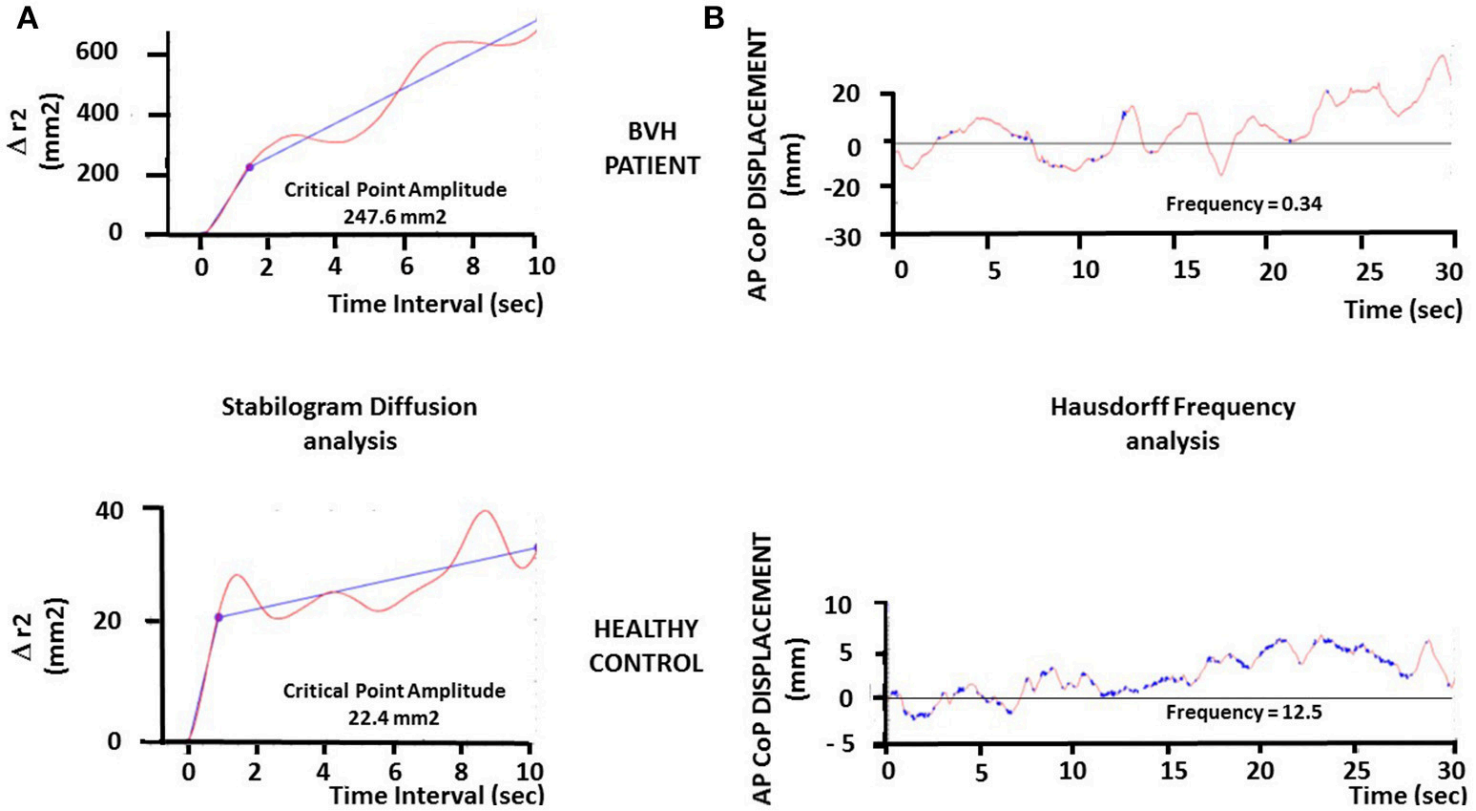
Illustration of the stabilogram-diffusion analysis and fractal analysis in one representative BVH patient and one representative healthy subject. **(A)**. Stabilogram-diffusion analysis provides a linear-linear plot of mean square antero-posterior CoP displacement vs. time interval. Planar stabilogram plots show the critical point as the intersection of the two regression lines performed on the raw CoP displacements data. The critical point provides the critical time interval (abscissae: in seconds) and the critical mean square displacement (ordinates: Δr^2^, in square millimeters). The critical time interval is similar in both the patient (upper graph) and the healthy subject (lower graph), while the critical mean square displacement is strongly increased in the BVH patient compared to the control (247.6 vs. 22.4 mm^2^). **(B)** Fractal analysis applied to the antero-posterior CoP displacements of the BVH patient (upper graph) and the healthy control (lower graph), illustrating the points in the samples (Hausdorff dimension) that are not correlated each other (in blue on the plots). These uncorrelated points illustrate the stochastic process in posture regulation, i.e., the events in the stabilogram which do not induce postural corrections (open loop or automatic control). The plots show a higher number of Hausdorff points in the control compared to the BVH patient, indicating a better postural stability in the control subject.

The CoP trajectories were studied also as one-dimensional and two-dimensional random walks, according to stabilogram-diffusion analysis [see (26), for details]. The displacement analysis of the CoP trajectories was carried out by computing the square of the displacement between all pairs of points separated by a specified time interval Δt, then averaged over the number of Δt of the recording session, and repeated for increasing values of Δt. The analysis provides a unique plot of the mean square CoP displacement (Δr^2^) vs. Δt (cf Figure 2A). The planar stabilogram-diffusion plots exhibit a short-term and a long-term region distinguishable on the basis of the coordinates of a critical point defined as the intersection point of the two curves fitting to these two regions. It is assumed that the spatio-temporal coordinates of this critical point approximate the region over which posture control switches from open-loop to close-loop control mechanisms. This method is particularly relevant to extract from the raw posturography data several parameters directly related to the steady-state behavior of quiet standing, or to the functional interactions with the neuromuscular mechanisms involved in the maintenance of upright stance. The amplitude of the critical point, expressed in mm^2^, is a good estimate of the limits over which posture is necessary corrected by feedback mechanisms to avoid fall.

The fractal analysis is based on fundamental concepts and principles from statistical-mechanics. It is aimed at determining if two consecutive points in the stabilogram are correlated, i.e., linked by a causal relationship (CoP is moving forward because of a previous backward displacement: feedback correction; close-loop control mechanism), or if these points are not correlated (random CoP trajectory, stochastic process: open-loop control mechanism). We have calculated the number of the Hausdorff points in each stabilogram, i.e., the number of sampling points in the CoP trajectories that are not correlated each other, and the mean frequency of these points in each visual condition for the CoP displacements in the AP and ML directions. The Hausdorff frequency parameter provides another estimate of posture stability. It allows to approximate the mean time-interval during which the patient remains stable without doing any postural correction. Higher the Hausdorff frequency, shorter the mean time-interval, and more stable the subject.

### Statistical analysis

Four parameters describing body sway during quiet standing were analyzed: the global score of posture control given by the Postural Instability index (PII), the Spectral Power Density (SPD) expressed in the frequency domain of the visual system (0.05–0.5 Hz frequency part), the amplitude of the critical point (CP amplitude) and the frequency of the Hausdorff points accounted by the stochastic process of posture control. To measure the effects of visual condition on posture control, i.e., darkness, visual fixation, visual pursuit with low (saccades) and high (smooth pursuit) flicker frequencies, the four postural parameters were analyzed using a 4-way ANOVA followed by a *post-hoc* analysis with the Tuckey test (Stateview II software). Results were considered significant for *p* < 0.05.

## Results

The ANOVAs performed on each of the posturography parameters pointed to significant differences between the BVH patients and the control group. The values were [*F*_(1, 40)_ = 12.54; *p* < 0.05] for the Postural Instability Index, [*F*_(1, 40)_ = 59.71; *p* < 0.001] for the SPD recorded in the visual frequency part, [*F*_(1, 40)_ = 68.45; *p* < 0.0001] for the amplitude of the critical point, and [*F*_(1, 40)_ = 70.50; *p* < 0.0001] for the frequency of the Hausdorff points.

The Figures 1B,C illustrates the wavelet transform applied to the stabilograms of one representative BVH patient examined in the four different visual conditions, and of one healthy control tested in darkness. Compared to the 3D map of the healthy subject, the BVH patient tested in the same visual condition exhibits a higher Postural Instability Index (PII: 3.48 vs. 1.32), and spends much more energy in the low frequency range (Log SPD = 94.5 vs. 72.40) to realize the postural task (Figure 1B). The figure shows also that both visual fixation and visual pursuit of a target, whatever the flicker frequency (strob 1: 4.5 Hz, slow eye movement; strob 2: 1.20 Hz, saccades), induce in the BVH patient a strong decrease of the PII. On the other hand, the spectral power density is strongly reduced compared to the task performed in total darkness, and particularly in the low frequency range of body sway (Figure 1C).

The Figure 2 illustrates the stabilogram-diffusion analysis (Figure 2A) and the fractal analysis (Figure 2B) performed on the CoP displacements of another representative BVH patient (upper graphs) compared to another healthy control (lower graphs) examined in darkness. It can be seen that the amplitude of the critical point in the BVH patient is strongly increased (247.6 mm^2^) compared to the control subject (22.4 mm^2^) (Figure 2A), indicating that the control subject shifts to close-loop control mechanisms for much more lower CoP displacements (~ 4.7 mm) than the control (~ 14.7mm). Moreover, the Hausdorff frequency is much higher in the healthy control (12.5 Hz) compared to the BVH patient (0.34 Hz) (Figure 2B). These values correspond to mean time-intervals of posture stability without postural corrections every 80 and 2,900 ms for the control (stable) and the patient (unstable), respectively.

The ANOVA performed on the Postural Instability Index (PII) of the BVH patients showed that visual condition constituted the main fixed effects responsible for the sources of variation among subjects [*F*_(3, 60)_ = 11.98, *p* < 0.00001]. This parameter was significantly decreased in the three visual conditions (Strobe 1: *p* < 0.0001, Strobe 2: *p* < 0.004, and Gaze fixated: *p* < 0.001) compared to darkness. The more destabilizing condition was observed in darkness (PII = 2.88 ± 1.19) whereas the lowest value was found in the Strobe 1 condition (PII = 1.69 ± 0.77). No significant differences were found between Fixation and Strob 1, and Fixation and Strob 2 in the patients. For comparison, the control group showed a significantly lower PII value in darkness compared to the BVH patients (PII = 1.97 ± 0.61; *P* < 0.006). However, the PII was not significantly modified with vision of a space-fixed target or eye movements in the healthy participants.

Rather similar findings were found for the three other parameters tested in the patients' group. The ANOVAs performed on the SPD in the visual frequency range, the amplitude of the critical point, and the Hausdorff frequency showed that visual conditions constituted the main fixed effects responsible for the variations among subjects, with [*F*_(3, 57)_ = 11.75, *p* < 0.0001], [*F*_(3, 57)_ = 5.63, *p* < 0.01], and [*F*_(3, 60)_ = 9.5, *p* < 0.00001], respectively. These three parameters were significantly different in all visual conditions compared to darkness, at highly significant levels. The SPD and the amplitude of the critical point were significantly decreased in gaze fixation, Strob 1 and Strob 2 conditions compared to darkness, while in the same time the Hausdorff frequency was significantly increased. The highest values were always observed in darkness (Log SPD = 85.61 ± 10.59; critical point amplitude = 328.3 ± 213.5 mm^2^), whereas the lowest values were found in the Strobe 1 condition (Log SPD = 76.48 ± 9.52; critical point amplitude = 100.23 ± 157.2 mm^2^). The opposite pattern was seen for the Hausdorff frequency, with the highest value in Strob 1 (1.13 ± 0.63 Hz) and the lowest in darkness (0.59 ± 0.36 Hz). By contrast, the visual conditions did not change so drastically the postural performance of the control group. No significant differences were found between darkness and the other visual conditions regarding the frequency of the Hausdorff points evaluated from the AP stabilogram; this parameter was improved only during the strob 2 condition (saccades) and from the ML stabilogram (*p* < 0.02). The SPD in the visual frequency part was not statistically modified in the ML direction, but it was significantly increased in darkness compared to the other visual conditions for the AP stabilogram. The amplitude of the critical point was significantly decreased in all visual conditions compared to darkness (*p* < 0.0001).

**Figure 3:**
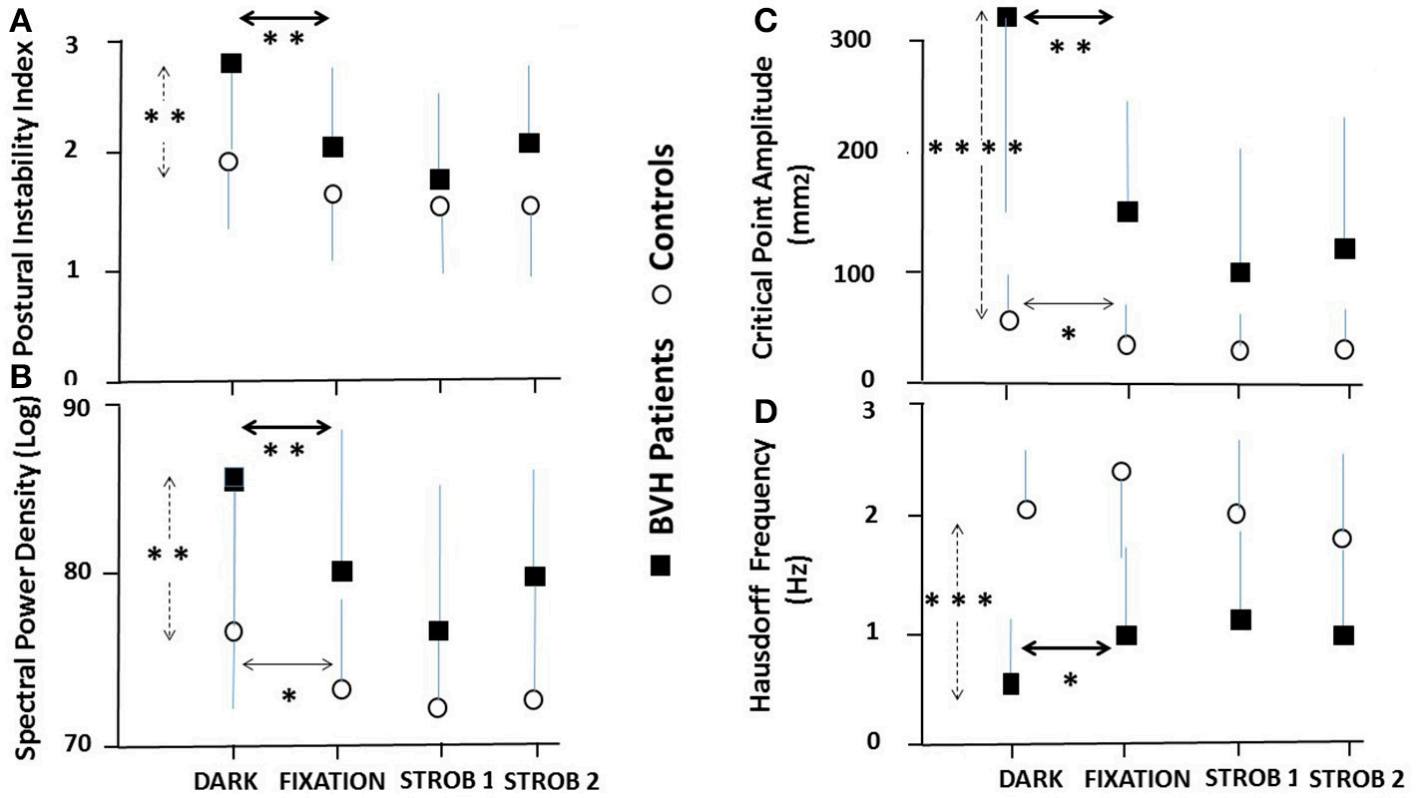
Effect of visual input and eye movements on posture stability in bilateral vestibular hypofunction patients and healthy participants. Mean results recorded in the population of BVH patients (*N* = 21: filled squares) and the control group (*N* = 21: open circles) in the four experimental visual conditions during quiet standing. **(A)** Mean postural instability index (±SD) derived from the wavelet transform recorded in darkness, during vision of a space-fixed target, eye pursuit of the sinusoidally moving visual target in the strob 1 condition (slow eye movements) and strob 2 condition (saccades). The vertical dashed arrow indicates significant differences in total darkness between the patients and the controls, while the heavy and light horizontal arrows show significant differences between darkness and the three other visual conditions in the patients and the controls, respectively. **(B)** Mean spectral power density (±SD) in the visual system frequency part (0.05–0.5 Hz), expressed in decimal logarithm and derived from the wavelet analysis. Same conventions as in **(A)**. **(C)** Mean amplitude (±SD) of the critical point calculated with the stabilogram-diffusion analysis. Same conventions. **(D)** Mean Hausdorff frequency (±SD) derived from the fractal analysis. The frequency was evaluated by dividing the number of uncorrelated points by the recording time. A higher frequency indicates a better body stabilization. Note that all four parameters point to a better postural stability when BVH patients fixate a visual target, do slow eye movements or saccades by comparison with quiet standing in darkness. In contrast, the different visual conditions do not modify so drastically the postural parameters in the control group compared to the patients. ^*^p < 0.05; ^**^p < 0.01; ^***^p < 0.001; ^****^p < 0.0001.

The Figure 3 summarizes the mean results recorded in the whole population of BVH patients and in the control group for the Postural Instability Index (PII: Figure 3A), the SPD in the visual frequency band (SPD: Figure 3B), the Amplitude of the Critical Point (CP amplitude: Figure 3C), and the Frequency of the Hausdorff Points (Hausdorff Frequency: Figure 3D). Compared to the test performed with eyes open in total darkness (dark), all four quantified postural parameters are significantly modified, attesting that posture stability was significantly improved when the patients fixated a stable space-fixed target (fixation), when they followed the moving target with smooth pursuit (strob 1 condition), and when performing saccadic eye movements (strob 2 condition). The control group exhibited postural improvement limited only to two postural parameters: the SPD in the AP direction, and the critical point amplitude, even though it remains at very low values in all visual conditions.

The comparison of the effects of visual condition on the AP and ML stabilograms in the BVH patients and in the control group is illustrated in the Table 1. The mean values (±standard deviation) of the SPD in the visual frequency band and of the Hausdorff points frequency are provided. Table 1 shows similar changes in both the AP and ML stabilograms for the BVH patients, with significantly reduced SPD values (*p* < 0.01) and significantly increased Hausdorff frequency (*p* < 0.01) during fixation, strob 1 and strob 2 conditions compared to darkness. Taken together, the results point to posture stability improvement in both directions when visual stimuli or eye movements are present. By contrast, the visual conditions do not change so drastically the postural performance of the healthy participants. Significant differences are observed only for the SPD of the AP stabilogram between darkness and the other visual conditions, and between darkness and strob 2 condition (saccades) for the Hausdorff frequency evaluated from the ML stabilogram (*p* < 0.05).

**Table 1:** Comparison of the effects of the visual condition on the spectral power density and the Hausdorff frequency for the AP and ML stabilograms recorded in the BVH patients, and in the control group.

	**Spectral power density (Log)**		**Hausdorff frequency (Hz)**	
	**AP CoP displacements**	**ML CoP displacements**	**AP CoP displacements**	**ML CoP displacements**
**BVH Patients**				
Dark	85.61 (10.50)	67.95 (17.35)	0.59 (0.36)	1.21 (0.80)
Fixation	79.78 (9.25)	60.92 (13.75)	1.00 (0.72)	2.01 (1.70)
Strob 1	76.48 (9.52)	57.23 (11.97)	1.13 (0.63)	2.64 (2.14)
Strob 2	79.68 (8.90)	57.21 (12.14)	1.07 (0.73)	2.91 (1.85)
**Controls**				
Dark	76.41 (5.90)	59.61 (7.25)	2.06 (0.50)	2.23 (0.53)
Fixation	72.81 (6.51)	57.65 (8.25)	2.62 (0.61)	3.06 (0.88)
Strob 1	71.38 (7.15)	55.63 (7.58)	2.09 (0.71)	2.79 (0.76)
Strob 2	72.48 (7.44)	56.83 (9.12)	1.71 (0.80)	3.08 (0.67)

*The mean spectral power density (decimal Log ± SD) evaluated in the visual frequency part (0.05–0.5 Hz) and the mean Hausdorff frequency (±SD) are given for each of the four visual conditions (dark, fixation, strob 1 condition: slow eye movements, and strob 2 condition: saccadic eye movements) for CoP displacements recorded in the antero-posterior (AP) and mediolateral (ML) directions*.

## Discussion

It is common to say that vestibular a-reflexic patients are strongly impaired without vision, because visual cues constitute strong extra-vestibular signals substituting to the lack of vestibular information in day life situations. Our results confirm this general statement since the comparison with healthy sex- and age-matched controls points to highly significant differences between the two populations for the tests performed in darkness.

The original finding of the present study is that posture control is differentially affected by the visual conditions in the BVH patients and the healthy participants. In the control group, the global postural score is not significantly modified with fixation or eye movements compared to darkness (mean PII around 2), and the Hausdorff frequency remains at high levels (around 2 Hz), corresponding to stable body positions every 0.5 s whatever the visual conditions. The amplitude of the critical point remains very low in all conditions, in the range 30–60 mm^2^, corresponding to postural corrections by the close-loop control mechanisms for CoP displacements as low as 5–8 mm. And the SPD in the low visual frequency part is significantly reduced with fixation and eye movements compared to darkness. Taken together, these data indicate that fixation of a stable space-fixed target, voluntary pursuit of a moving target and eye-tracking with saccades slightly improve the postural performance of the healthy participants mainly by reducing the energy cost to control body sway, and by increasing the efficacy of the close-loop control mechanisms.

By contrast, the BVH patients tested in darkness have significantly higher PII (2.88) and critical point amplitude (328.30 mm^2^), spend more energy to control posture (higher SPD in the visual frequency part), and show lower Hausdorff frequency (corresponding to stable positions every 1.63 s). All the values recorded point to a strong degradation of posture control without vision in the BVH patients. The results clearly show however that visual input provided by fixation of a stable space-fixed target, by voluntary pursuit of a moving target with smooth pursuit, or during saccades strongly improved posture stability in the BVH patients compared to their postural performance recorded in total darkness. In these visual conditions, the patients' postural performance attested by the PII becomes quiet similar to that recorded in healthy controls. Moreover, the SPD, the critical point amplitude and the Hausorff frequency become much closer to the controls, suggesting that the BVH patients use all the sensorimotor inputs provided by their eye movements as substitution processes to improve posture control.

Many studies investigated the visual contribution to postural stability in healthy subjects [(27–29), for reviews]. Visual fixation of a stationary target was reported to improve postural stability by decreasing body sway, and it has been proposed that the CNS can interpret the eye movement inputs to gain positional information (2). These authors have distinguished two modes of visual detection of body displacements. The ocular mode, based on the retinal slip (motion of the target on the retina), is very unlikely a mechanism playing a role in our experimental conditions. Indeed, retinal slip is minimized both by the smooth pursuit system and the vestibulo-ocular reflex, if any. The extra-ocular hypothesis is more likely because this second mode is based either on ocular motor efference copy signals or proprioceptive reafferences from the extra-ocular muscles. Reduction of postural sway during fixation suppression of the nystagmus in patients with vestibular neuritis supports the role of ocular motor signals rather than that of pure visual cues elicited by retinal slip for the visual control of body sway (30).

The effect of eye movement on posture stability in healthy subject shows contrasting results in the literature. Schulmann et al. (31) concluded that tracking eye movements has a negative effect on balance, a result confirmed by Glasauer et al. (8), while smooth pursuit and saccades improved balance (32). A better postural control was also reported by Stoffregen et al. (7) and Rougier et al. (6) when subjects performed saccadic eye movements, confirming a more ancient study (5). The data suggest that saccadic and tracking eye movements have fundamentally different effects on posture; tracking with slow eye movements affect body sway but not saccades since vision is suppressed during saccadic eye movements (28, 29, 33). Of particular interest are the anatomo-histological findings showing that eye muscle fibers implicated in slow phase eye movements like smooth pursuit and nystagmus are non-twitch fibers characterized by a rich contain of neuromuscular spindles, while twitch fibers implicated in saccadic eye movements would be less richly endowed with muscle spindles (34). Our data do not show however significant differences in posture stability during saccadic or slow eye movements in our BVH patients, even though the best postural stability was always seen during tracking with smooth pursuit. In our experimental conditions, the extra-retinal signals are the major source of visual information during saccades, tracking and fixation of a stable target, suggesting that the same motor ocular factors are involved. That could explain why we found no changes in postural sway between these experimental conditions in the BVH patients.

How explaining however the discrepancy regarding the opposite effects of slow eye movements on posture control between healthy controls and BVH patients? The different postural describers used to investigate balance control could be one explanation. Most of the studies reported above have computed very simple describers (length and area of CoP displacements) that are not sensitive enough to describe precisely how posture is regulated. They do not take into account the energy cost to control posture and they ignore possible postural strategies. Our more functional parameters cannot explain however why opposite results were obtained with the same conventional postural describers used in the previous studies. The different populations tested (patients vs. controls) could be another explanation. How slow eye movements could destabilize posture in healthy subjects and unilateral vestibular loss patients, and have stabilizing effects in BVH patients. One hypothesis supported by our data is that total loss of vestibular functions induces a more power anchoring on eye proprioception compared to patients with a remaining labyrinth or healthy subjects. The BVH patients would use more than controls their eye muscle proprioceptive afferents, and/or the efference copy derived from the ocular motor command. Another one is to consider the effects of dual-tasking reported in the literature on posture control. Keeping quiet standing with a concomitant visual task is the illustration of a simple dual-task in which the visual task (fixation or pursuit of a visual target) interferes with the postural task (stand quietly). Such interactions have been reported since a long time with dual-task paradigms combining postural tasks and cognitive tasks, in which either posture or cognitive performance were altered due to the necessity to share the attentional resources between the two tasks (35). In healthy adults, it was proposed that attention is more focused on the cognitive task and, as a consequence, posture stability is improved because posture control is shifted to a more automatic mode of posture control (21, 36)]. On the contrary, deficits in the allocation of attention with aging or pathology have been suggested to explain the less efficient balance performance in dual-task conditions (21, 37, 38). The present study showed that our chronic BVH patients are more stable and spend less energy to control their posture in the visual tasks with fixation, slow eye movements and saccades. The mean age of the patients (62.9 years) being still outside the old senior population, the secondary visual tasks could indeed contribute to improve their posture stability more than in healthy controls. Indeed, not only the PII and the SPD in the visual frequency part were strongly reduced, but the Hausdorff frequency was increased, indicating that the BVH patients are more frequently stable compared to the dark condition without secondary visuo-motor task. Moreover, the amplitude of the critical point was reduced significantly, a result suggesting that the close-loop mechanisms of posture control are evoked for smaller CoP displacements.

Taken together, our data confirm that BVH patients have poor postural control in total absence of visual cues, and support that new idea that visual detection of body sway is one mechanism used by the BVH patients to improve their posture stability. The present findings strongly suggest that extra-ocular proprioceptive re-afferences or copy of the eye motor command are very likely involved, and over-used in a compensatory sensorimotor substitution process. On the other hand, when the BVH patients are in dual-task conditions, they shift to a more automatic mode of posture control that contributes also to improve their postural performance.

## Limits of the study

Our population of BVH patients is heterogeneous and includes patients with remaining vestibular functions regarding the otolith organs. Even though all the patients were submitted to vestibular rehabilitation therapy, the rehabilitation programs were not the same for all patients, and the history of the disease was also different in terms of duration and feeling of the handicap. The impact on the quality of life was attested in our BVH patients with regard to balance control and visual contribution to posture regulation, but not concerning the psycho-affective dimension. We tested the BVH patients on stable support only, not on foam or unstable support, another experimental condition that could provide useful information on proprioception contribution to posture control.

## Author contributions

ML, CVN, and MT conceived and designed the experiments. ML, AT, SH, and ND performed the experiments. ML wrote the paper.

### Conflict of interest statement

The authors declare that the research was conducted in the absence of any commercial or financial relationships that could be construed as a potential conflict of interest.
